# Exploring productivity hotspots in the Precambrian biosphere

**DOI:** 10.1098/rstb.2024.0103

**Published:** 2025-08-07

**Authors:** Eva E. Stüeken

**Affiliations:** ^1^ School of Earth and Environmental Sciences, University of St Andrews, St Andrews KY16 9TS, UK

**Keywords:** primary productivity, energy limitation, nutrient limitation, hydrothermal vents, estuaries, early Earth

## Abstract

Earth’s earliest biosphere was likely limited by metabolic energy. Nutrient limitation, which imparts a strong control on productivity today, only began with the origin of oxygenic photosynthesis *ca* 3 billion years ago (Ga). This contribution builds upon these concepts to explore how the spatial distribution of primary producers evolved across this transition from energy- to nutrient-limited. While on the modern Earth hotspots of primary productivity are centred around deep-marine upwelling zones and estuaries, preliminary calculations suggest that the early chemotrophic biosphere may have been fuelled by hydrothermal injections of H_2_ and Fe^2+^, making volcanically active basins at least 2–8 times more productive relative to background. The rise of oxygenic photosynthesis in the Neoarchean likely enabled the expansion of primary producers into freshwater habitats, which provided nutrients by weathering and perhaps boosted biological diversification. In the Proterozoic, when the deep ocean was nutrient-depleted, primary productivity was probably clustered around estuarine settings, where it may have been enhanced by a factor of 3–25. In conclusion, the spatial distribution of primary producers has likely evolved over the past 4 billion years. Accounting for this trend may help identify biogeochemical limits and opportunities in future studies of the early Earth and other habitable words.

This article is part of the discussion meeting issue ‘Chance and purpose in the evolution of biospheres’.

## 1. Introduction

**Figure 1.**
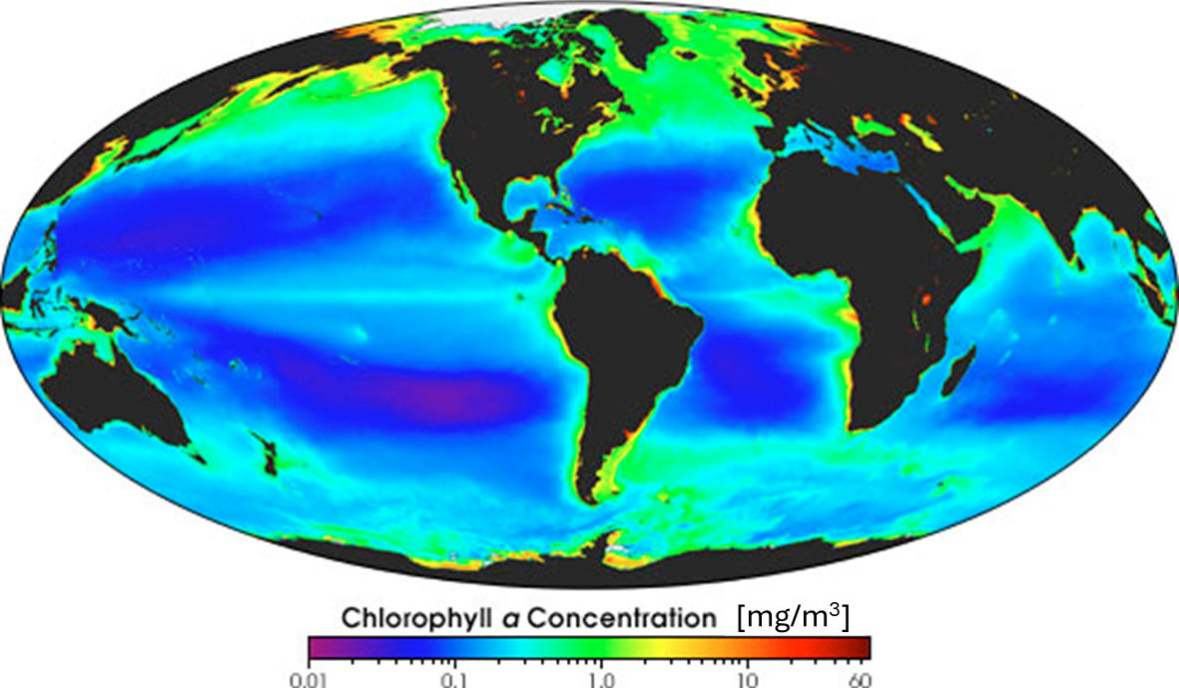
Chlorophyll *a* concentration in the surface ocean in units of mg m^−3^ inferred from satellite observations. Taken from the NASA Earth Observatory project (https://earthobservatory.nasa.gov/images/4097/global-chlorophyll).

Perhaps one of the most iconic images of our time is a satellite photo of Earth’s surface with superimposed chlorophyll *a* concentration (figure 1). This image is not only a demonstration of humanity’s technological capabilities, but it also illustrates better than anything else what drives primary productivity on the modern Earth. Chlorophyll *a* is a photosynthetic pigment that captures solar photons and thus effectively serves as an antenna in oxygenic phototrophs, including all cyanobacteria and plants, i.e. today’s dominant primary producers. Its distribution across the Earth’s surface reveals that marine primary productivity is elevated in regions of coastal and equatorial upwelling and near estuaries of major rivers.

The main driver of this pattern is the supply of nutrients from the deep ocean and from continental runoff. In the modern ocean, a number of elements display so-called ‘nutrient-type’ profiles in the open water column, where surface waters are highly depleted and deep waters below the surface mixed layer are enriched [1, and references therein]. Such profiles arise from quantitative consumption of the nutritious elements in surface waters, where photosynthetic primary producers are most active, followed by remineralization at depth as dead biomass sinks out of the surface mixed layer and is oxidatively degraded. As a corollary, deep waters stimulate biological productivity in regions where upwelling feeds these nutrients back into the photic zone [2]. Similarly, productivity in estuaries is stimulated by nutrient runoff from weathering of rocks and from the degradation of biomass on land, as well as anthropogenic nutrient input on the modern Earth [3,4]. The global marine chlorophyll *a* distribution (figure 1) therefore attests to the nutrient-limited photosynthetic lifestyle that powers the modern biosphere and that has created biological ‘hotspots and hot moments’ [5], also referred to as ‘ecological control points’ [6], in regions and time periods of elevated nutrient input.

However, in more general terms, life can be limited not only by the supply of nutrients but also by the supply of energy (electron donors and/or electron acceptors). The origin of oxygenic photosynthesis at *ca* 3 Ga [7–9] and its rise to ecological dominance has placed the biosphere into a position where it is effectively tapping into an unlimited energy supply [10]. This is because widely abundant H_2_O is serving as the electron donor, coupled to electron acceptors in the form of excited photosystems that merely require exposure to sunlight. This explains why hotspots of primary productivity occur in regions of maximum nutrient supply today (figure 1).

By contrast, previous studies concluded that prior to the origin of oxygenic photosynthesis, energy rather than nutrients may have been in short supply [10]. Estimates of the productivity of a pre-photosynthetic biosphere driven by H_2_ or Fe^2+^ (table 1) fall up to four orders of magnitude short of the modern value of 3.8 × 10^15^ mol C yr^-1^. These estimates support the hypothesis of a stepwise increase in primary productivity from the origin of life to the origin of anoxygenic phototrophy and finally to the origin of oxygenic phototrophy. The results further imply that ecological hotspots for the early biosphere may have been clustered around point sources of metabolic energy and not necessarily around point sources of nutrients.

**Table 1.** Published estimates of net primary productivity (NPP) for specific metabolisms on the early Earth in units of moles of carbon per year. For comparison, modern NPP = 3.8 × 10^15^ mol C yr^-1^*.*

reference	H_2_-drivenmethanogenesis	H_2_-drivenphototrophy	Fe^2+^-drivenphototrophy	global avg.Archean	global avg.Proterozoic
Kharecha *et al*. [11]	1.86−3.61 × 10^12^	1.86−9.38 × 10^13^	1.9 × 10^13^		
Canfield *et al*. [12]	3.4 × 10^12^	2.9 × 10^13^	1.4−4.7 × 10^14^^a^		
Ward *et al*. [10]^b^	2.43 × 10^11^	2.43 × 10^12^	3.2 × 10^11^^a^	0.3−2.5 × 10^12^	10^13^−10^15^
Planavsky *et al*. [13]				4.8 ± 1.3 × 10^12^	8.5 ± 3.5 × 10^13^
Crockford *et al*. [14]^c^				10^10^−10^14^	10^12^−10^14^

^a^ Subtracting the contribution of H_2_-driven phototrophy from the value for overall anoxygenic phototrophy.

^b^ Converted from mol e^−^ yr^-1^ to mol C yr^-1^ by dividing by 5, according to Ward *et al.* [10].

^c^ Scaled to modern value of *ca* 10^15^ mol yr^-1^.

From the Neoarchean onwards, oxygenic phototrophs likely became the dominant primary producers, meaning that the biosphere transitioned from energy-limited to nutrient-limited [13,14]. Planavsky *et al.* [13] estimated a global average productivity of 8.4 ± 3.5 × 10^13^ mol C yr^-1^ for the Proterozoic, later supported by Crockford *et al.* [14]. This estimate is higher than most NPP estimates for the early Archean, prior to oxygenic photosynthesis (table 1), but still nearly 100 times lower than the modern value, illustrating the impact of nutrient starvation during the Proterozoic, when the deep ocean was anoxic, inhibiting the accumulation of large nutrient reservoirs. Therefore, ‘hotspots and hot moments’ in the Proterozoic biosphere would have been defined by excess supplies of nutrients, but without being able to rely on a deep-marine nutrient supply along upwelling zones that characterizes the modern Earth (figure 1). In the following, I will explore transitions in ecological hotspots over Earth’s history under different metabolic regimes, accounting for overarching trends in the geological evolution of our planet.

## 2. Proposing hydrothermally active basins as early Archean productivity hotspots

The earliest biosphere was likely chemotrophic and limited by the supply of chemical electron donors and acceptors. The most plausible chemotrophic metabolisms of primary producers under Archean conditions, in the absence of free O_2_, would have been methanogenesis (coupling H_2_ oxidation to CO_2_ reduction) and associated secondary metabolisms such as fermentation [10–12]. Metabolisms based on sulfur (reduction of S^0^ or SO_4_^2−^ coupled to H_2_ or CH_4_ oxidation) and nitrogen oxides (NO_x_ reduction coupled to oxidation of H_2_, Fe^2+^ or CH_4_) would have made minor contributions. NPP driven by methanogenesis would have been constrained by volcanic outgassing of H_2_ gas and the rate of H escape from the upper atmosphere into space. Both Kharecha *et al.* [11] and Canfield *et al.* [12] assumed that atmospheric H_2_ diffuses from the atmosphere into the ocean, where it is rapidly consumed. Hence according to these models, even in an H_2_-limited methanogenic biosphere, most primary production would have occurred near the ocean–atmosphere interface. Also S^0^, SO_4_^2−^ and NO_x_ would have been supplied by atmospheric sources, specifically photochemical conversion of volcanogenic SO_2_ gas [15] and lightning-driven oxidation of N_2_ [16,17]. Therefore, these minor metabolisms are also thought to have contributed to a surface-ocean dominated biosphere.

Once anoxygenic photosynthesis evolved, the dependence on sunlight would have further exacerbated this spatial trend. Both Kharecha *et al.* [11] and Canfield *et al.* [12] derived their calculations of global Fe^2+^-driven phototrophic productivity from estimates of deep-marine Fe^2+^ concentrations, paired in one case with an assumed modern upwelling flux [11] and in the other case with the average C:P:Fe stoichiometry of anoxygenic phototrophs and the assumption that those organisms were ultimately limited by upwelling of P (rather than Fe) into the surface ocean [12]. According to these models, the early Archean biosphere would thus have resembled the modern biosphere in that it was concentred in the surface ocean. With the onset of anoxygenic photosynthesis, life may even have migrated towards regions of Fe^2+^ (and perhaps P) upwelling into the photic zone and created similar clusters as today (figure 1), although it is important to recognize that upwelling regimes in the Archean, prior to large-scale growth and emergence of continental crust, may have been distributed differently around the globe, and ocean mixing timescales may have varied under shorter day lengths [18]. While these arguments for upwelling-driven productivity are plausible, I propose here that hydrothermally active basins acted as point sources of H_2_, Fe^2+^ and other nutrients, which may have created ‘hotspots and hot moments’ on the early Archean Earth that were independent from upwelling regions.

Canfield *et al.* [12] distinguished between subaerial and submarine volcanism (including hydrothermalism) as sources of H_2_ and found the latter to constitute *ca* 16–46% of the total H_2_ flux. Catling & Kasting [19] revisited this calculation and proposed *ca* 30% submarine H_2_ input. Both studies used data from the modern world to constrain these proportions, and there are reasons to assume that the marine serpentinization-derived H_2_ flux would have been higher in the past. First, the early Archean likely had less exposed land mass [20] and likely a higher proportion of oceanic crust relative to continental crust [21]. Second, trace element data from chemical sedimentary rocks in the Archean indicate more intense submarine hydrothermal activity, possibly due to a hotter mantle [22]. Third, a larger fraction of the oceanic crust was ultramafic in composition [23] and thus conducive to serpentinization [24,25]. Lastly, there is increasing evidence that the early Archean mantle had a lower oxygen fugacity and that the modern fugacity was only reached around the time of the Paleoproterozoic Great Oxidation Event in response to sediment subduction [26,27]. Hence the composition of gases released from early Archean vents were perhaps more reducing. Therefore, H_2_ production in the marine realm was likely higher than it is today—both in absolute and relative terms. Likewise, the flux of Fe^2+^ sourced from hydrothermal vents would have been elevated. A strong hydrothermal Fe^2+^ flux has long been implicated in genetic models of banded iron formations [28]. Kump & Seyfried [29] calculated Fe^2+^ concentrations of *ca* 80 mM for Archean vent fluids in the presence of very low seawater sulfate concentrations and reduced hydrostatic pressure. Hence the two major metabolic substrates for early Archean primary producers (H_2_ and Fe^2+^) probably displayed their highest concentrations in hydrothermal environments.

**Figure 2.**
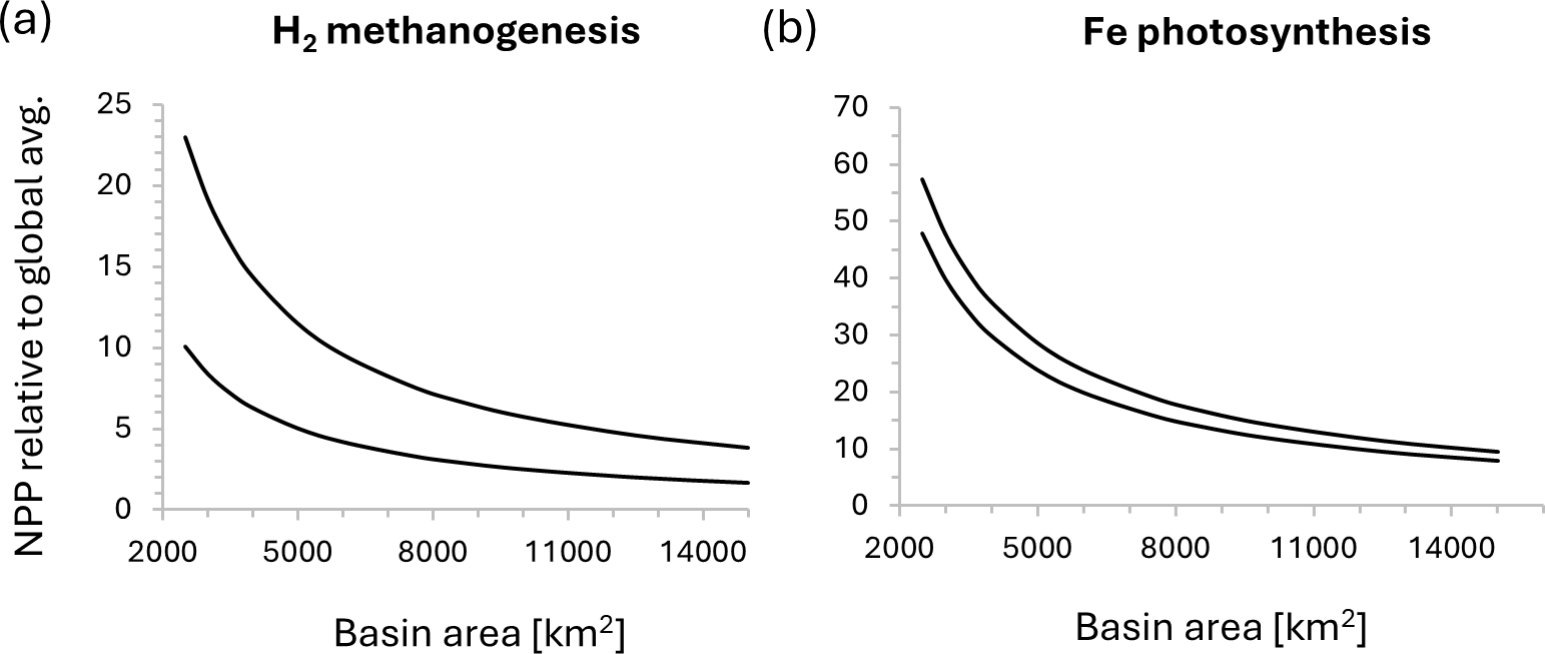
Enhancement in net primary productivity (NPP) relative to global average NPP in small hydrothermally active basins with water fluxes similar to the modern Guaymas Basin. Global average NPP is taken from Kharecha *et al.* [11] for the same metabolisms. (a) Enhancement in H_2_-based autotrophic methanogenesis. (b) Enhancement in Fe^2+^-based anoxygenic photosynthesis.

To quantitatively explore the potential of hydrothermal venting to enhance NPP locally within a volcanically active basin, I turn to the modern Guaymas Basin in the Gulf of California, which hosts a series of hydrothermal seeps and vents along two spreading axes (known as the Northern and Southern Trough), offset by a transform fault [30,31]. The entire basin is *ca* 240 km long, 60 km wide (area *ca* 14 400 km^2^) and 2 km deep [32], where the two hydrothermally most active trough regions (Southern Trough: *ca* 20 km × 4 km; Northern Trough: *ca* 40 km × 6 km) [30] make up less than 10% by area. Hence the hydrothermal influence on the Guaymas Basin is distributed across a relatively small water mass, especially if only the region immediately above the two spreading axes is considered. Previous workers estimated a hydrothermal fluid flow of *ca* 10−12 m^3^s^-1^ [33]. Multiplying this flow rate with Fe^2+^ concentrations of 80 mM as proposed for the Archean [29] would yield an Fe^2+^ flux of 2.5−3.0 × 10^10^ mol yr^-1^. This number can be converted into Fe^2+^-stimulated productivity by using the C:Fe stoichiometry of 1:4 of anoxygenic photosynthesis [11]. NPP stimulated by this flux would thus have been 6.3−7.6 × 10^9^ mol C yr^-1^. Scaling this number to the area of the Guaymas basin makes it comparable with global average estimates of NPP for this metabolism (table 1), which can in turn be normalized to surface area with the conversion factor 1 mol yr^-1^ = 0.00374 molecules cm^-2^ s^-1^ [11]. The Fe^2+^-driven NPP from Kharecha *et al.* [11] is used here as a global average reference point, because counter to the higher estimates of Canfield *et al.* [12] their calculations do not assume that life is limited by phosphorus (further discussed below). Their results are also in agreement with more recent estimates by Ozaki *et al.* [34] for this metabolism. This calculation shows that Fe^2+^-driven productivity within the Guaymas Basin, under these hypothetical Archean conditions, would have been at least 8−10 times higher than global background, depending on the surface area over which this hydrothermal fluid is distributed (figure 2). In a scenario where hydrothermal fluids create a focused flux immediately above the active spreading centres, the enhancement could be greater than a factor of 40−60. For comparison, modern upwelling zones show NPP enhancements by a factor of *ca* 10−100 relative to the open ocean (figure 1), highlighting that the enhancement calculated a Guaymas-sized hydrothermally active basin in the Archean would have been significant. If the Fe^2+^-driven NPP from Ward *et al.* [10] were used as a global average reference, the hydrothermal effect would be *ca* 100 times higher. Also worth highlighting in this context is that Fe-fluxes from modern hydrothermal vents in the Southern Ocean and South Pacific have been invoked to explain local pulses of primary productivity in the overlying photic zone [35,36]. The effect of the hydrothermal flux on productivity ultimately depends on the buoyancy of the hydrothermal fluid, which may vary widely, but these modern observations show that hydrothermal nutrients are not necessarily lost by dilution and can thus have a significant regional impact on the biosphere.

A similar calculation can be performed with H_2_. The concentration of H_2_ in endmember black smoker fluids can be less than 1 mM [37], but as pointed out by Catling & Kasting [19], this concentration likely underestimates the true flux of H_2_ from hydrothermal processes into the ocean (see also [38]). Accounting for serpentinization [19] would lead to a corrected average H_2_ concentration of *ca* 8 mM. For comparison, concentrations of up to 19 mM have been measured from modern vents influenced by serpentinization [39]. Assuming that 1 mol of H_2_ leads to 0.1 mol C-fixation [11], shows that H_2_-fuelled methanogenesis would also have been enhanced at least two to fourfold and possibly 10−20-fold in hydrothermally active basins like the Guaymas Basin in the Archean (figure 2). Such basins may therefore have served as productivity hotspots, akin to upwelling zones today.

## 3. Transitioning from energy- to nutrient-limitation

A corollary of the NPP models for the early Archean is that the biosphere was not limited by nutrients, including P and N. Recent evidence supports this view and may be consistent with a transition towards a more nutrient-limited regime in the Neoarchean. Regarding phosphorus, estimates of Archean seawater concentrations, based on geochemical data and models, range over more than five orders of magnitude from <0.01 µM to >1000 µM (see compilation by Boden *et al.* [40]). Intriguingly, high estimates are derived from models that assume a hydrothermal P input [41]. Hydrothermal circulation can leach P from bedrock [42], and it may enhance P-recycling from organic matter—a process that was otherwise probably suppressed under anoxic conditions in the Archean [43]. Hydrothermal remobilization of ammonium from organic-rich sedimentary strata is documented from the modern and Archean ocean [44,45], and it is conceivable that P was remobilized in the same manner. In addition to recycling fixed nitrogen, hydrothermal vents could also have catalysed the abiotic reduction of N_2_ and nitrogen oxides to ammonium [46,47], where the latter were likely supplied by lightning reactions and volcanic heating in the atmosphere [16,17,48]. Flux estimates of these abiotic sources of bioavailable nitrogen (10^9^−10^12^ mol yr^-1^) are *ca* 10−1000 times smaller than modern biological N_2_ fixation, which is the major source of nitrogen to the biosphere today (10^13^ mol yr^-1^) [49]. These fluxes may have been sufficient to sustain early life if NPP was a few orders of magnitude lower than today (table 1). Therefore, hydrothermal settings on the early Archean Earth may not only have been point sources of metabolic energy substrates but perhaps they also contributed to the proposed excess supply of phosphorus and nitrogen prior to the origin of photosynthesis [10].

The genomic record can shed additional light on how these nutrient cycles evolved as the biosphere became more complex. As noted earlier, the invention of oxygenic photosynthesis has been dated to *ca* 3 Ga, based on phylogenetic reconstructions [7,9,50]. This age is supported by geochemical data [51]. Cyanobacteria then further expanded in the Neoarchean, from *ca* 2.7 Ga onwards [7]. Intriguingly, the enzyme nitrogenase may have emerged around a similar time as oxygenic photosynthesis in the Mesoarchean [52]. Also this inference is consistent with the geochemical record [53,54]. Nitrogenases further expanded across the tree of life in the Neoarchean [52], concurrent with cyanobacterial expansion [7,55]. Meanwhile, phylogenetic reconstructions of P-uptake enzymes found that the oldest enzymes, which date back to the early Archean, were those that are adapted to high phosphate concentrations today [40]. Enzymes optimized for progressively lower phosphate levels evolved later in the Archean. Collectively, these records may thus reflect a transition towards a biosphere that became increasingly nutrient-stressed as photosynthesis evolved and liberated primary producers from the constraints of energy limitation. Nutrient limitation would have drawn down the marine phosphate reservoir and perhaps triggered the invention and radiation of nitrogenase enzymes. As discussed below, this trend perhaps pushed ‘hotspots and hot moments’ in the biosphere towards continental habitats.

## 4. Continental boost of biodiversity in the Neoarchean

With the invention of oxygenic phototrophs, the dependence on hydrothermally supplied Fe^2+^ and H_2_ would have lessened, perhaps enabling primary producers to expand into a wider range of habitats. Importantly, the late Meso- to Neoarchean is also thought to be the time when land masses emerged above sea level at large scale [20,56,57]. This event would have created numerous habitats in freshwater settings, perhaps ideal for organisms that were capable of using H_2_O as their energy source and took advantage of locally sourced P from weathering [58] as the marine P reservoir dwindled (see above).

**Figure 3.**
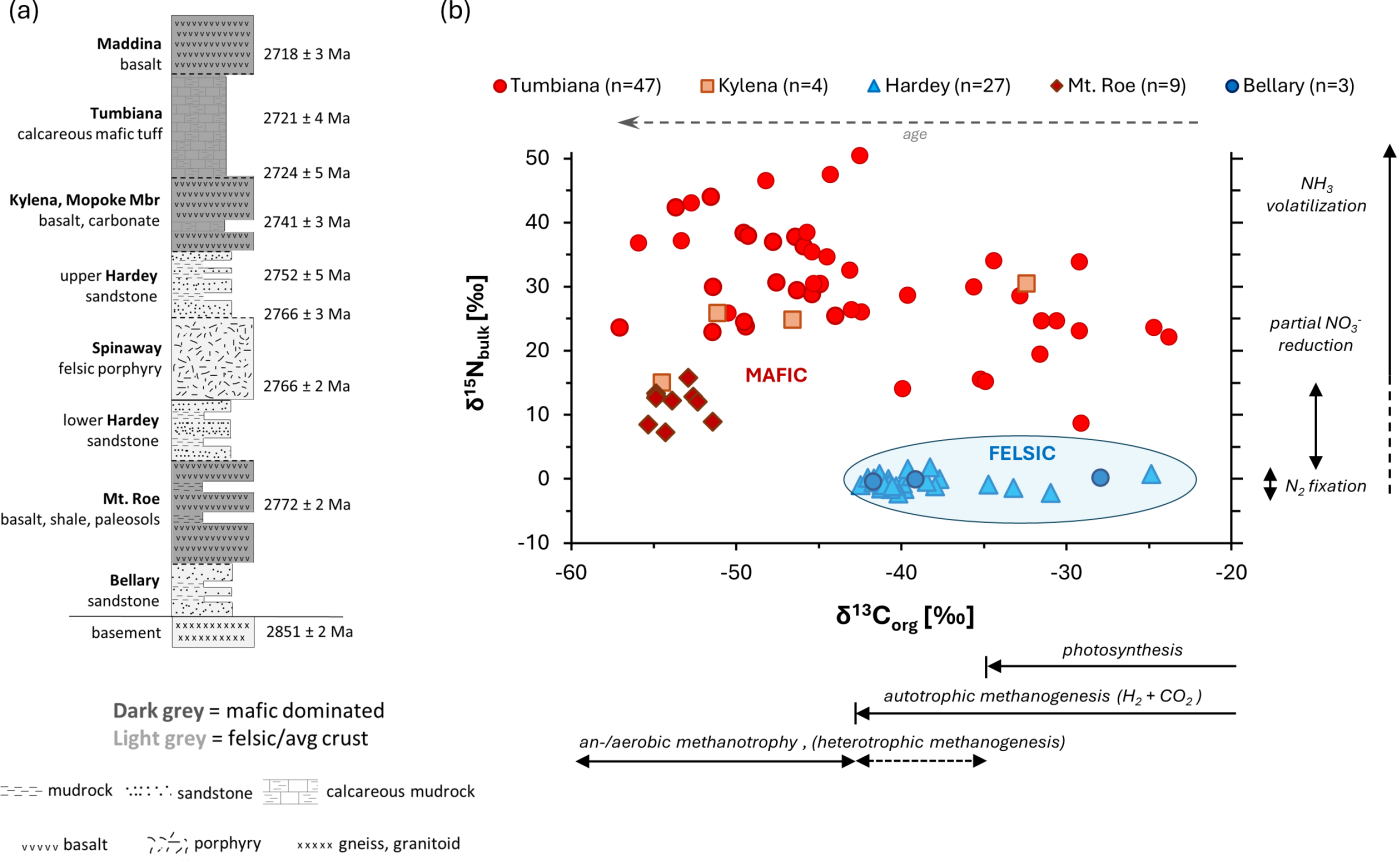
(a) Stratigraphy of the Neoarchean Fortescue Group in Western Australia; (b) organic carbon and nitrogen isotope data from the Fortescue strata (after Stüeken *et al.* [66]). Results show strong dependence of isotopic data on environmental conditions*.*

Increasing environmental diversity may also have created niches for biological diversification. An example of this diversity can be seen in the Neoarchean Fortescue Group in Western Australia (2.8−2.7 Ga). Here, a package of non-marine sedimentary strata is preserved with alternating felsic and mafic mineralogies, depending on the lithologies of the eroding source rocks [59]. The sediments were likely deposited in rivers, fluvial overbank deposits and lakes [60–63]. Lakes deposited within mafic bedrock in the Tumbiana Formation are enriched in carbonate with stromatolites up to 2 m in size [62]. Unusually high nitrogen isotope values from these rocks have been interpreted as a combination of redox processes [64] and NH_3_ degassing at high pH [65]. By contrast, the felsic-hosted settings likely had circum-neutral pH. Interestingly, organic carbon isotopes differ markedly and systematically between mafic- and felsic-hosted environments in the Fortescue Group (figure 3), independent of age [66]. This wide range of values cannot be explained by a single metabolism and instead suggests a range of metabolic strategies that were variably enhanced or suppressed by environmental conditions. This dataset is therefore evidence for the effect of terrestrial habitat diversity on biological diversity and perhaps underscores the importance of crustal emergence for biological innovation.

## 5. Proterozoic productivity hotspots at ocean margins

From the Proterozoic onwards, non-marine habitats were probably widely populated. Phylogenetic data suggest that photosynthetic eukaryotic organisms emerged in low-salinity environments [67], further attesting to the importance of continental niches for biological innovation and diversification. The Proterozoic deep ocean was probably depleted in phosphate, which kept NPP in the surface ocean at low levels compared with today [13,14,68]. By contrast, at least some Proterozoic freshwater environments and estuarine settings show evidence of P input and enrichment [69,70], perhaps making them hotspots for NPP at that time. This idea was conceptually tested by Laakso & Schrag [68], whose model results indeed showed that weathering fluxes of nutrients would have played a more important role relative to upwelling in sustaining NPP during the Proterozoic, such that the biosphere was probably concentrated in coastal habitats. Importantly, the global extent of coastal habitats and their nutrient load may have been limited in the Proterozoic due tectonic quiescence and prolonged supercontinentality [71]. It is therefore critical to identify regions that deviated from nutrient-depleted background conditions.

An example of a phosphorus-rich coastal habitat in the Proterozoic that hosted a diverse ecosystem of prokaryotic and eukaryotic organisms is the Diabaig Formation in northwestern Scotland [72,73]. Once interpreted to represent an ancient lake [74], more recent analyses have revealed a marine connection with minor but persistent seawater influx, consistent with an estuarine setting [75]. Microbial mats, likely of cyanobacterial origin, are preserved on numerous bedding planes [76], and eukaryotic microfossils have been described from discrete horizons [73]. Phosphate concretions confirm the presence of a consistent P supply [70], possibly linked to the Grenville orogeny. Nitrogen was perhaps fixed locally but recycled from microbial mats into the water column during diagenesis, making it available to non-N_2_-fixing organisms [77].

**Figure 4.**
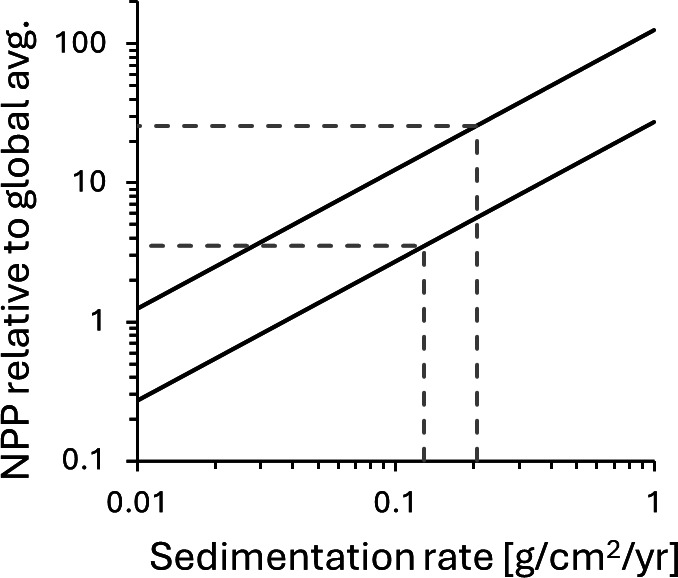
Enhancement in net primary productivity (NPP) in the 1.0 Ga Diabaig Formation in NW Scotland relative to global average NPP derived by Planavsky *et al.* [13]. The two solid black lines mark upper and lower bounds, accounting for uncertainties in total organic carbon content of the Diabaig rocks [75] and in the global average NPP value (8.4 ± 3.5 × 10^32^ mol yr^-1^). The dashed grey lines highlight sedimentation rates in a modern estuarine overbank setting [78]*.*

Primary productivity in the Diabaig Formation can be estimated from published records of total organic carbon content [75] and estimates of sedimentation rate. I further assume that the preserved biomass represents *ca* 8 +/− 2% of the original biomass prior to diagenetic degradation, as proposed for the Proterozoic [13]. This is probably an upper limit, considering that these sediments display mud cracks [75] and were thus exposed to atmospheric O_2_. The results can be compared with the proposed global average Proterozoic NPP of 8.4 ± 3.5 × 10^13^ mol C yr^-1^ [13], after normalizing the latter to surface area with the conversion factor of 1 mol yr^-1^ = 0.00374 molecules cm^-2^s^-1^ [11]. The results (figure 4) show that NPP in the Diabaig estuary may have been 3−25 times higher than the global average for a sedimentation rate similar to modern overbank deposits. A riverine overbank environment is a plausible analogue, considering the high mud content in the Diabaig rocks. An NPP enhancement of this magnitude is similar to that seen in modern upwelling zones (figure 1) and supports the notion that estuarine settings linked to catchments with erosive nutrient sources may have represented productivity hotspots during the Proterozoic, when upwelling regions were perhaps less attractive.

## 6. Conclusions

The origin of oxygenic photosynthesis marked a turning point in the history of life, because it freed primary producers from the constraints of electrochemical energy sources such as Fe^2+^ and H_2_. Instead, the biosphere became limited by nutrients, in particular phosphorus and nitrogen, whose spatial distribution creates significant heterogeneity in primary productivity on Earth today. In particular, marine upwelling zones and estuaries characterized by high levels of nitrate and phosphate influx have become known as ‘hotspots’ in modern ecology. Prior to the emergence of oxygenic photosynthesis, when methanogens and anoxygenic phototrophs were likely the main primary producers, preliminary calculations suggest that hydrothermally active basins may have supported at least 2−8 times higher productivity relative to the global average. Hence, ecological hotspots may not necessarily have been tied to coastal upwelling regions. Once oxygenic phototrophs evolved, the dependence on hydrothermal input of Fe^2+^ and H_2_ would have waned, perhaps enabling organisms to expand into a wider range of environmental niches, including continental settings. Here, the diversity of habitats created by differing bedrocks may have boosted biological diversification. An increase in primary productivity with the onset of oxygenic photosynthesis perhaps led to a shrinkage of the marine phosphate reservoir and the invention of biological N_2_ fixation, consistent with phylogenetic and geochemical records. The transition towards nutrient limitation from the Neoarchean onwards may have pushed productivity hotspots to coastal environments and estuaries. Moving forward, the hypotheses presented in this study will require more thorough quantitative testing with three-dimensional models and additional field evidence. A number of geological and environmental parameters should be considered in greater detail, such as changes in the distribution and intensity of upwelling zones over Earth’s history [18]; the character of hydrothermal venting under a stagnant lid tectonic regime as it may have existed in the early Archean [79,80]; secular trends in the composition of continental crust and their effects on continental habitats and fluvial fluxes [81]; and the role of tectonic quiescence during the Proterozoic [71]. Considering how these parameters impact spatial trends and heterogeneities in nutrient sources through geologic time will enable us to more accurately reconstruct the trajectory of life on Earth.

## Data Availability

This article has no additional data.
